# Clinical features and treatment outcomes of septic arthritis due to *Mycobacterium massiliense* associated with intra-articular injection: a case report 

**DOI:** 10.1186/s13104-016-2245-6

**Published:** 2016-09-15

**Authors:** Haekyung Lee, Dohui Hwang, Minchul Jeon, Eunjung Lee, Taehyong Kim, Shi Nae Yu, Yongbeom Kim, Byung-ill Lee

**Affiliations:** 1 Division of Infectious Disease, Department of Internal Medicine, Soonchunhyang University Hospital, 59 Daesagwan-ro, Yongsan-gu, Seoul, Korea; 2 Department of Orthopedic Surgery, Soonchunhyang University Hospital, 59 Daesagwan-ro, Yongsan-gu, Seoul, Korea; 3 Department of Internal Medicine, Soonchunhyang University Hospital, 17 Soonchunhyang 6-gil, Dongnam-gu, Cheonan, Chungcheongnam-do Korea

**Keywords:** *Mycobacterium massiliense*, Non-tuberculous mycobacteria, Septic arthritis, Intra-articular injection, Case report

## Abstract

**Background:**

There are increasing reports on nosocomial *Mycobacterium massiliense* infection, but septic arthritis and osteomyelitis because of that microorganism is rare. This report focuses on the clinical aspects of *M. massiliense* arthritis outbreak concurrent with soft tissue infection.

**Case presentation:**

An outbreak of septic arthritis among patients who had been injected at a single clinic occurred in South Korea between April and September 2012. This may be associated with repeated injection of triamcinolone contaminated with *M. massiliense.* Nine of the Korean patients visited our hospital complaining of painful swelling of the knees. During treatment course, patients are suffered from soft tissue abscess around the injection site. Acid-fast bacilli culture for infected tissue was positive in five patients, and polymerase chain reaction for non-tuberculous mycobacteria was positive in four patients. They were treated with antibiotics, repeated arthroscopic surgeries, incision and drainage for a long time. All patients were eventually cured but three patients have suffered from a decreased range of motion.

**Conclusion:**

Early clinical suspicion and microbiological diagnosis are key factors in reducing morbidity since septic arthritis with *M. massiliense* manifests late after the injection and treatment of it is a laborious process.

## Background

Non-tuberculous mycobacteria (NTM) are ubiquitous in nature widely found in soil, wastewater, and other materials [1]. *Mycobacterium massiliense*, a rapidly growing mycobacteria (RGM), was first isolated from the sputum and bronchoalveolar lavage of a patient with pneumonia in 2004 [2]. As a human pathogen, it can cause various diseases including respiratory infections, abdominal infections, skin and soft tissue infections, especially local infections and abscesses after various surgeries or other procedures [3].

Recently, reports on nosocomial NTM outbreaks in various clinical situations have significantly increased. Because *M. massiliense* cases involving joints are scarce, we will focus on the clinical features, therapeutic experiences and prognosis of *M. massiliense* arthritis.

## Case presentation

Two hundred forty four (19.3 %) patients of the 1264 who had visited the primary local clinic from April 2012 to September 2012 had one or more injections. Twenty-seven cases were reported to the Korea Institute of Drug Safety and Risk Management (KIDS) as adverse drug events by the physician at the clinic [4].

Our hospital is a 750-bedded tertiary medical center and major academic teaching hospital which is about 16 km away from the clinic.

The nine Korean patients who had been injected at the clinic visited our hospital because of painful swelling of the knees. They all had a history of multiple intra-articular injections of the steroid mixed with analgesics in the same local clinic. They complained of fever (89 %), redness (56 %), heating sensation (45 %), discharge (34 %) of infected site, and decreased range of motion (67 %). Eight of the patients were female and one patient was male. The mean age was 58.8 years (range 49–71 years). The median time from the injection date to the onset of symptoms was 4.7 weeks (range 2–9 weeks).

Joint fluid or infected tissues were obtained by needle aspiration or arthroscopic biopsy for Gram and Acid-fast bacilli (AFB) stain, bacterial and AFB culture, and polymerase chain reaction (PCR) for *Mycobacterium tuberculosis* (MTB) and NTM. Specimens were stained with the Auramine-rhodamine and Ziehl-Neelsen stain for AFB and plated onto Ogawa medium, BACTEC MGIT 960 (Becton, Dickinson and Company), blood agar, and MacConkey agar.

In our hospital setting, MTB/NTM PCR is a real-time PCR which uses a primer that targets IS*6110* which is a MTB specific gene, and a primer and a Taqman probe that targets an internal transcribed spacer (ITS) for identifying mycobacteria at the species level. So this can detect MTB and *Mycobacterium* genus through a single reaction.

We sent the same specimens to the Korean National Tuberculosis Association. They identified NTM by PCR-restriction fragment length polymorphism (PCR-RFLP) analysis targeted to *rpoB* gene. They tested drug susceptibility by broth microdilution method, and reported minimum inhibitory concentration in accordance with Clinical and Laboratory Standards Institute guidelines.

Synovial fluid leukocyte was elevated in all patients (range 4770–28,800/μl), percentage of neutrophils was in the 10–95 % range, and higher than 65 % in four patients. AFB culture was positive in five patients, and PCR was positive for NTM in four patients. Result of AFB culture and PCR expressed as a positive or negative was same for all patients, except for one patient. AFB culture and MTB/NTM PCR of synovial fluid was not checked in one patient.

AFB stain of tissue obtained by arthroscopy was positive in two patients, AFB culture was positive in eight, and MTB/NTM PCR was positive in six. Microorganism was not identified in one patient. *M. massiliense* was finally detected in eight patients by using PCR-RFLP.

The antibiotics susceptibility test (Table 1) for *M. massiliense* was done in six isolates but was not checked in two isolates.

**Table 1:** Results of drug susceptibility test against *Mycobacterium massiliense*

Minimum inhibitory concentration (μg/ml) of antibiotics																		
Patient number	AK		CFX		CIP		CLA		DOX		IMP		MXF		TMP/ SMX		LNZ	
1	16	S	32	I	8	R	≤0.5	S	32	R	8	I	2	I	4/76	R	≤2	S
2	16	S	32	I	4	R	≤0.5	S	8	R	8	I	2	I	8/152	R	4	S
3	Not checked																	
4	16	S	64	I	16	R	≤0.5	S	16	R	16	I	4	R	16/304	R	8	S
5	16	S	16	S	8	R	≤0.5	S	>32	R	4	S	2	I	4/76	R	4	S
6	Not isolated																	
7	Not checked																	
8	8	S	32	I	8	R	≤0.5	S	>32	R	4	S	4	R	4/76	R	≤2	S
9	16	S	16	S	16	R	≤0.5	S	>32	R	8	I	8	R	8/152	R	4	S

*AK* amikacin, *CFX* cefoxitin, *CIP* ciprofloxacin, *CLA* clarithromycin, *DOX* doxycycline, *IMP* imipenem, *MXF* moxifloxacin, *TMP/SMX* trimethoprim/sulfamethoxazole, *LNZ* linezolid, *S* susceptible, *I* intermediate, *R* resistant

Magnetic resonance imaging was performed in eight of the nine patients. It showed a moderate to large joint effusion, intra-meniscal signal intensity change, diffused synovial enhancement and thickening, and chondral lesion with subchondral bone changes. In two of the patients, osteomyelitis was suspected due to increased bone marrow edema.

All patients were treated with combination of antibiotics for a long period, repeated arthroscopic irrigations and debridements. Three patients underwent arthroscopic adhesiolysis and six patients underwent brisement to improve contracture of the knee joint. All of them suffered from accompanying skin and soft tissue abscesses around injection sites (knee, thigh, lower leg, buttocks and back) during treatment progress. Five patients were repeatedly admitted to our hospital due to recurrence of septic arthritis and soft tissue abscess despite maintenance of antibiotics. Incision and drainage for seven patients and excision for one was done to remove abscess. Patients underwent from one up to seven times of surgery.

Most arthroscopic findings revealed that synovium was hypertrophic and hyperemic because of inflammatory changes and multiple granulation tissues were filled-up. Damage of cartilage seemed to proceed slowly. Intra-articular fat pad changes were seen because of fat tissue involvement with necrosis and discharge. The majority of abscesses tended to appear on the subcutaneous fat layer around injection sites. They appeared like masses and contained a turbid, yellowish discharge inside enclosed by a thickened capsule.

Empirical antibiotics were intravenous (IV) vancomycin plus anti-pseudomonal third generation cephalosporin (ceftazidime) to cover *Staphylococcus aureus* especially for methicillin resistant *S. aureus* or *Pseudomonas aeruginosa* which are important pathogens related with the procedures. After confirming that PCR was positive for NTM, some patients were treated with oral clarithromycin, IV amikacin and imipenem for RGM. After the culture confirmed the existence of *M. massiliense*, we maintained the combination of oral clarithromycin, IV amikacin and imipenem for six patients. For two patients whose drug susceptibility were not analyzed and another patient whose tissue culture revealed no organism, we treated them with the same antibiotics based on the epidemic circumstances. Complications associated with antibiotics did occur like dizziness in seven patients, hearing deterioration in three, tinnitus in two and otalgia in one. We could not keep amikacin due to high tone hearing loss in four patients and acute kidney injury in one patient. One patient complained of nausea and vomiting because of the imipenem.

The average duration of medical treatment among the nine patients except that of empirical antibiotics was 382.9 days (range 174–776 days). Details on the characteristics of patient, treatment course and outcome are presented in Table 2. All patients were eventually cured but three patients kept on complaining of a decreased range of motion.

**Table 2:** Patient characteristics, treatment and outcome

Patient number	Sex/age	Past history	Injection count	Incubation period (weeks)^a^	Antibiotics and surgeries^b^	Outcome (range of motion)
1^e^	F/54	Breast cancer	Six	4	Cefazolin (2 days) Ceftazidime (8 days) + vancomycin (3 days) → ceftazi dayime + teicoplanin (2 days) Amikacin (68 days) + imipenem (81 days) + clarithromycin (184 day) Arthroscopic irrigation and debridement (X2) Brisement Readmission due to knee abscess Imipenem (8 days) + clarithromycin (137 days) Arthroscopic irrigation and debridement Incision and drainage of knee abscess	Rt. 0–120 Lt. 0–120
2	F/51	Hypertension	Six	4	Cefazolin (2 days) Ceftazidime (7 days) + vancomycin (7 days) Amikacin (30 days) + imipenem (110 days) + clarithromycin (171 days) Arthroscopic irrigation and debridement (X2) Incision and drainage (X3) of back, buttock abscesses Arthroscopic irrigation and debridement Brisement (X2) Arthroscopic adhesiolysis Readmission due to thigh, calf abscesses Imipenem (12 days) + clarithromycin (92 days) Incision and drainage of abscesses	Full
3	F/56	Diabetes mellitus	Two	6	Cefazolin (4 days) Ceftazidime (3 days) + vancomycin (3 days) Amikacin (34 days) + imipenem (80 days) + clarithromycin (174 days) Arthroscopic irrigation and debridement (X3) Arthroscopic adhesiolysis Brisement Open excision of back abscess	Full
4	F/65	Diabetes mellitus	Multiple (above eight)	7	Cefazolin (1 day) Ceftazidime (2 days) + vancomycin (2 days) Amikacin (29 days) + imipenem (81 days) + clarithromycin (399 days) Arthroscopic irrigation and debridement (X2) Excision of thigh abscess Incision and drainage of back abscess	Full
5	F/59	Subclinical hypothyroidism	Four	3	Cefazolin (1 day) Ceftazidime (3 days) + vancomycin (3 days) Amikacin (27 days) + imipenem (81 days) + clarithromycin (100 days) Arthroscopic irrigation and debridement (X2) Arthroscopic adhesiolysis Brisement Readmission due to recurrence of septic arthritis and buttock abscess Imipenem (25 days) + clarithromycin (160 days) Arthroscopic irrigation and debridement Incision and drainage of buttock abscess	Full
6^f^	F/63	Hyperlipidemia Osteoarthritis	Five^c^	2	Ceftazidime (3 days) + vancomycin (3 days) Amikacin (29 days) + imipenem (46 days) + clarithromycin (321 days) Arthroscopic irrigation and debridement	Rt. 0–125
7^g^	M/61	Psoriasis Septic arthritis	Two	5–9 (assumed^d^)	Ceftazidime (1 day) + vancomycin (1 day) Amikacin (27 days) + imipenem (233 days) + clarithromycin (253 days) Arthroscopic irrigation and debridement (X5) Incision and drainage (X5) of popliteal fossa, both buttock, thigh and knee abscesses Brisement Excision of thigh, knee abscesses	Rt. 0–125 Lt. 0–120
8	F/71	Hypertension Diabetes mellitus Herniated intervertebral disc	Multiple (above six)	3	Cefazolin (1 day) Ceftazidime (3 days) + vancomycin (3 days) Amikacin (16 days) + imipenem (71 days) + clarithromycin (117 days) Arthroscopic irrigation and debridement Excision and revision of necrotic wounds of neck, back Debridement of back abscess Arthroscopic irrigation and debridement Incision and drainage of neck, back abscesses Readmission due to abscesses of multiple sites Imipenem (34 days) + clarithromycin (98 days) Arthroscopic irrigation and debriment Incision and drainage of neck, back, buttock and leg abscesses Outpatient clinic follow up due to abscesses of multiple sites Imipenem (15 days) + clarithromycin (62 days) + levofloxacin (28 days) Readmission due to Rt. leg, back abscesses Imipenem (25 days) + clarithromycin (29 days) Incision and drainage, excision of abscesses Outpatient clinic follow up due to abscesses of multiple sites Clarithromycin (77 days) + linezolid (57 days) Amikacin (20 days) + imipenem (20 days) + clarithromycin (294 days) Clarithromycin (99 days) + doxycycline (99 days)	Full
9^h^	F/49	Osteoarthritis	Multiple	5–8 (presumed^d^)	Amikacin (6 days) + imipenem (67 days) + clarithromycin (67 days) Arthroscopic irrigation and debridement Brisement Cefoxitin (48 days) + clarithromycin (164 days) Arthroscopic irrigation and debridement Incision and drainage of knee abscess Clarithromycin (88 days) + linezolid (14 days) Readmission due to recurrence of septic arthritis Cefoxitin (13 days) + amikacin (13 days) + clarithromycin (182 days) Readmission due to knee abscess and recurrence of septic arthritis Cefoxitin (38 days) + amikacin (38 days) + clarithromycin (178 days) + [ciprofloxacin (9 days) → levofloxacin (148 days)] Excision of knee abscess	Full

^a^ Incubation period means time between exposure to first injection and appearance of the first symptoms

^b^ We list antibiotics before and surgeries later with a number in the order, and assign new number if patient readmitted. We indicate duration of antibiotics and number of surgeries in parenthesis. The alphabet “d” in parenthesis following antibiotics is abbreviation for day

^c^ The patient had four injections by the original clinic and one injection by a different private clinic after the onset of symptoms

^d^ Patients could not recall the exact onset of the symptoms and recalled on a monthly basis rather than weekly. So we set incubation period from the first week to last week on the basis of their memory

^e^ Vancomycin was stopped due to drug fever and was replaced by teicoplanin

^f^ Small abscesses of both knees appeared when the patient followed up observations in an outpatient setting. We recommended her admission but she refused

^g^ The patient was discharged against medical advice and did not return due to financial limitations

^h^ The patient was treated with isoniazid, rifampin, ethambutol and pyrazinamide for 6 days at another hospital before visiting our hospital. We changed imipenem to cefoxitin because of nausea, vomiting. The patient was further readmitted twice due to postoperative pus discharge and knee abscess recurrence in spite of maintenance of oral clarithromycin and ciprofloxacin replaced with levofloxacin

## Discussion

*Mycobacterium abscessus* complex is RGM characterized by growth in solid agar media within seven days and subclassified into three closely related subspecies of *M. abscessus*, *M. massiliense*, and *M. bolletii* [1]. *M. massiliense* has an identical 16S ribosomal RNA (rRNA) sequence that of *M. abscessus* but can be differentiated from *M. abscessus* by using *hsp65, rpoB, sodA, recA* genes and 16S–23S rRNA ITS sequences [2]. Because of the close relationship between *M. abscessus* and *M. massiliense*, it is possible that *M. massiliense* had been misidentified as *M. abscessus* in previous studies. In one study, *M. massiliense* constitutes a large proportion of RGM isolates previously identified as *M. abscessus* [5].

*Mycobacterium massiliense* has a different pattern of erythromycin ribosomal methylase (*erm*) gene from *M. abscessus*, and showed a good response to the clarithromycin-containing regimen [1]. Because the susceptibility to clarithromycin of *M. massiliense* differs from *M. abscessus*, distinguishing between the two organisms is clinically important.

Recently, there have been increasing reports on *M. abscessus* complex soft tissue infections in immunocompetent people. This is resistant to disinfectants, and easily forms biofilm which can cause post-surgical and post-procedural infections. Contaminated materials (reusable needles or syringes, diluents for injection, wash water for injector, and inappropriate disinfectant) play an important role in nosocomial infections [4]. Soft tissue infections caused by *M. massiliense* have been reported associated with pacemaker pocket infections [6], intramuscular injections [7], arthroscopic, laparoscopic [3] and cosmetic procedures [8].

This report describes the first outbreak of septic arthritis due to *M. massiliense* associated with intra-articular injections administered at a local clinic, in Seoul, South Korea. Among the 244 patients who were injected at the clinic, 61 patients were participated in the analysis conducted by KIDS and Korea Center for Disease Control and Prevention [4] and 9 patients of the 61 were hospitalized in our hospital. All septic arthritis patients were injected with triamcinolone. The triamcinolone injection agent was prepared by mixing 0.5 ml of triamcinolone from a 1 ml container, 1 ml of saline from a 20 ml container and 1 ml of lidocaine from a 20 ml container. These containers were reused later. The nursing assistant injected the agent into multiple sites using a single syringe without wearing gloves. No microorganism was identified in the environmental samples, empty vials, injection medications, injection needles, syringes and alcohol swabs. However, the reuse of triamcinolone contaminated with *M. massiliense* from the clinic environment and repeated injection may have affected this post-injection outbreak. Insufficient sterilization (hand washing, wearing gloves, appropriate skin disinfection, soaking cotton balls in appropriate disinfectant but not boiled tap water, aseptic preparation of the injection agent, and procedure) have possibly contributed to an increase of the risk of infection. It was likely that the risk factors for septic arthritis including advanced age, comorbidities such as rheumatoid arthritis, osteoarthritis, and diabetes mellitus played a role in the *M. massiliense* infection. The cessation of outbreak was well correlated with a closure of the clinic [4].

Combination therapy of IV amikacin with cefoxitin or imipenem and an oral macrolide such as clarithromycin, azithromycin is recommended for *M. abscessus* by the American Thoracic Society/Infectious Diseases Society of America [9] and is applied for *M. massiliense* in many centers. However, treatment of it is an increasingly frequent challenge to clinicians, because of the extended treatment duration, use of multiple antimicrobial agents and drug related toxicities. Surgical debridement remains an important element of successful therapy especially for extensive disease, necrosis, or abscesses. Surgical excision or drainage combined with a backbone of clarithromycin for 3–6 months appeared to be the most suitable therapy for post-injection abscess [10].

Since septic arthritis with NTM manifests several days up to months after the injection, and empirical therapeutic approaches commonly used to treat soft tissue infections are not effective against NTM, early clinical suspicion and microbiological diagnosis are key factors in reducing morbidity [3].

## Conclusion

In our cases, the patients were treated with long term IV and oral antibiotics combined with repeated surgical interventions. The duration of the oral clarithromycin treatment ranged from 174 to 776 days. Patients underwent repeated surgeries up to seven times. Although *M. massiliense* is an emerging pathogen of soft tissue infection associated with procedure or surgery, septic arthritis and osteomyelitis rarely occur and its clinical information and therapeutic options are still not fully understood. We report this outbreak of septic arthritis by *M. massiliense* with characteristics of patients, clinical manifestations, laboratory and synovial fluid analysis, treatment duration and progress, highlight the difficulty in treating the *M. massiliense* infection and expect it to be a help in the diagnosis and treatment of septic arthritis caused by *M. massiliense*.

## Abbreviations

AFB: acid-fast bacilli
ITS: internal transcribed spacer
IV: intravenous
KIDS: Korea Institute of Drug Safety and Risk Management
*M. massiliense*: *Mycobacterium massiliense*
MTB: *Mycobacterium tuberculosis*
NTM: non-tuberculous mycobacteria
PCR: polymerase chain reaction
PCR-RFLP: PCR-restriction fragment length polymorphism
RGM: rapidly growing mycobacteria
rRNA: ribosomal RNA
*S. aureus*: *Staphylococcus aureus*
